# Follicular dendritic cell sarcoma associated with IgG4-related disease: a case report and literature review

**DOI:** 10.3389/fimmu.2026.1685017

**Published:** 2026-05-29

**Authors:** Yajuan Yao, Yunjing Bai, Guang Liu, Yu Du, Xiaoyu Long, Xiaohua Xu, Fan Wu, Xiaorong Jin

**Affiliations:** 1 Department of Rheumatism and Immunology, Seventh Medical Center of Chinese PLA General Hospital, Beijing, China; 2 Department of Pathology, Seventh Medical Center of Chinese PLA General Hospital, Beijing, China

**Keywords:** esophagus, follicular dendritic cell sarcoma, gastric, IgG4-related disease, liver

## Abstract

Follicular dendritic cell sarcoma (FDCS) is a rare malignant tumor involving lymph nodes and extranodal sites, classified into conventional and inflammatory pseudotumor (IPT)-like variants. The IPT-like variant predominantly involves the liver and spleen. Immunoglobulin G4-related disease (IgG4-RD) is a systemic immune-mediated disorder characterized by multiorgan involvement, whereas isolated esophageal and gastric involvement is exceedingly rare. Although FDCS may exhibit infiltration of IgG4-positive plasma cells, the coexistence of FDCS and IgG4-RD has not been previously reported. Herein, we describe a unique case of FDCS associated with IgG4-RD involving the liver, esophagus, and stomach. The patient initially presented with hepatic lesions and gastrointestinal ulcers, accompanied by elevated serum IgG4 levels and histopathological features suggestive of IgG4-RD. Following glucocorticoid and immunosuppressive therapy, gastrointestinal lesions improved; however, the hepatic lesion progressed and was subsequently confirmed as FDCS by histopathology and immunohistochemistry. This case highlights the diagnostic challenges posed by overlapping clinicopathological features between FDCS and IgG4-RD.

## 1 Introduction

Follicular dendritic cell sarcoma (FDCS) is a rare low-grade malignant tumor derived from follicular dendritic cells and classified among histiocytic and dendritic cell neoplasms (1). It can involve both lymph nodes and extranodal sites. The etiology and pathogenesis remain unclear but may be associated with EBV infection, Castleman disease, and autoimmune disorders. FDCS is categorized into conventional and inflammatory pseudotumor (IPT)-like variants. The IPT-like variant predominantly affects the liver and spleen.

Immunoglobulin G4-related disease (IgG4-RD) is a systemic immune-mediated disorder characterized by elevated serum IgG4 levels and distinctive histopathological features, including dense lymphoplasmacytic infiltration, storiform fibrosis, and obliterative phlebitis (2). Although IgG4-RD can involve the gastrointestinal tract, isolated esophageal and gastric involvement remains rare and diagnostically challenging. Previous studies have shown that FDCS may be accompanied by IgG4-positive plasma cell infiltration. However, the coexistence of FDCS with definitively diagnosed IgG4-RD has not been previously reported.

## 2 Case presentation

A 60-year-old male presented to our hospital with “a liver lesion for 10 days.” On April 7, 2014, a liver lesion was detected during a physical examination at an external hospital. The patient had no abdominal pain or distension, fever, jaundice, nausea, or vomiting. On April 17, 2014, for surgical treatment, the patient was admitted to the Department of Hepatobiliary Surgery of our hospital via the outpatient clinic with a diagnosis of “liver lesion”. Upon admission, the patient had stable mental status, normal sleep, good appetite, normal bowel and bladder functions, and no significant change in body weight.

## 3 History of past illness

The patient denied any history of chronic diseases such as hypertension, diabetes, or hepatitis. The patient underwent routine physical examinations in 1992 and 2005, during which liver lesions were detected. Both lesions were surgically resected with histopathological confirmation of inflammatory pseudotumor in each instance. Four years ago, the patient sustained a left tibial plateau fracture and underwent surgical treatment (details unspecified). The patient has a history of blood transfusion (specifics unknown) and denies any history of drug allergies.

## 4 Personal and family history

The patient’s personal and family histories were unremarkable.

## 5 Physical examination upon admission

Temperature 36.2 °C, pulse 86 bpm, respiratory rate 21 breaths/min, blood pressure 16/8 kPa. No enlarged lymph nodes were palpated. Cardiorespiratory examination revealed no abnormalities. The abdomen was flat, with surgical scars measuring approximately 20 cm in length below the bilateral costal margins and a 15 cm midline scar. The abdomen was soft, non-tender, and without rebound tenderness. The liver and spleen were not palpable below the costal margin. No shifting dullness was detected, and bowel sounds were normal. Joints showed no swelling or tenderness. No edema was observed in the lower extremities.

## 6 Laboratory examinations

Laboratory examinations showed tumor markers(alpha-fetoprotein (AFP), carcinoembryonic antigen (CEA), carbohydrate antigen 19-9 (CA19-9), CA72-4, CA50, CA242, CA125, and CA15-3), ANA profile, and ANCA were negative. White blood cell count, platelet count, liver/renal function, three immunoglobulins, and C-reactive protein (CRP) were within normal limits. Notable abnormalities included: erythrocyte sedimentation rate (ESR): 25 mm/h (normal < 20mm/h), Complement C3: 0.8 g/L(normal 0.9–1.8g/L), Hemoglobin(HGB): 101 g/L(normal 120–160 g/L), IgG4: 1590 mg/L (normal 80–1400 mg/L).

## 7 Imaging examinations

Contrast-enhanced abdominal CT (**Figure 1**) demonstrated a well-circumscribed, round-like hypodense lesion in the left hepatic lobe, measuring approximately 4.7 cm × 4.7 cm with peripheral enhancement and an apparent capsular sign. The gastric wall along the lesser curvature was markedly thickened and showed enhancement. In the left kidney, a round hypodense lesion and a round hyperdense lesion were noted at the lower pole, both without significant enhancement. No other abnormalities were identified in the pancreas, spleen, adrenals, or retroperitoneal lymph nodes. The impression was a malignant space-occupying lesion in the left hepatic lobe, marked gastric wall thickening requiring further evaluation, and left renal lesions suggestive of benign cysts.

**Figure 1:**
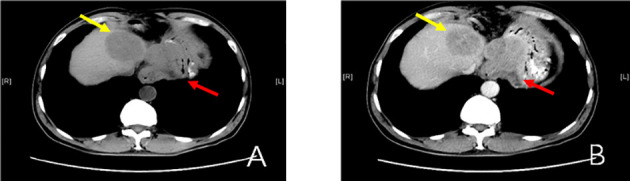
Multiple lesions in the liver and stomach seen on computed tomography. The slightly hypodense area in the left lobe of the liver indicated by the yellow arrow shows peripheral enhancement, and a capsule appears to be visible at the margin. The thickened gastric wall at the lesser curvature side of the stomach indicated by the red arrow shows enhancement.

Gastroscopy: Esophagus(**Figure 2A**): A protruding lesion with central ulceration was identified at the anterior wall of the lower esophagus, approximately 38 cm from the incisors. Stomach(**Figure 2B**): A giant ulcerative lesion was observed in the mid-upper body of the stomach along the lesser curvature, with rigid gastric wall and loss of peristalsis.

**Figure 2:**
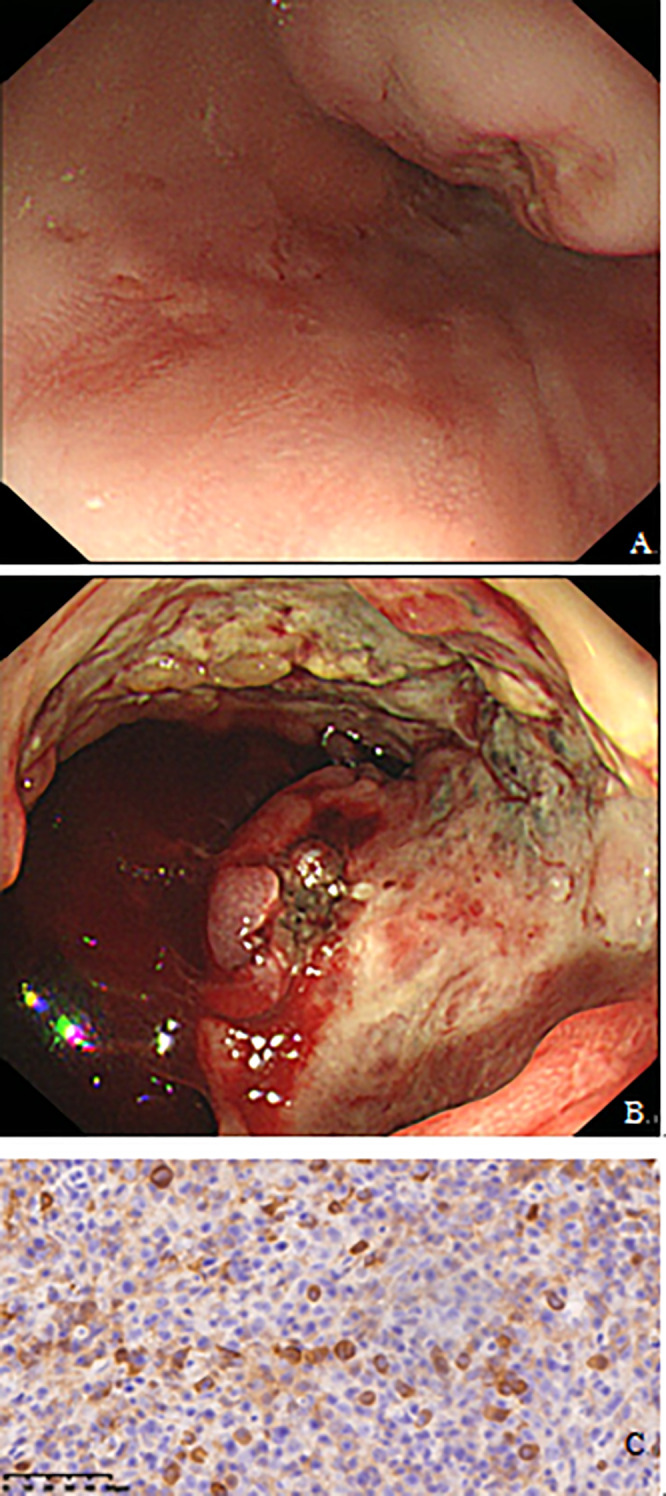
**(A)** Endoscopic view of the lower esophagus of a hard fixed lesion and a ulcer. **(B)** Endoscopic view of giant stomach ulcer. **(C)** IgG4-positive cells seen in esophageal and stomach biopsies.

Pathology: Gastric lesser curvature biopsy: Degenerative necrotic tissue and fibrous connective tissue with infiltrates of neutrophils, lymphomononuclear cells, and abundant plasma cells. Esophageal biopsy (37 cm): Squamous-columnar junction mucosa with severe chronic inflammation, erosion, and fibrotic proliferation in the lamina propria, accompanied by dense plasma cell infiltration.

Immunohistochemistry (IHC)(**Figure 2C**):Focal IgG4-positive plasma cells >50/high-power field (HPF),IgG4/IgG ratio >30%. Diagnostic note: IgG4-RD cannot be excluded based on histopathological and IHC findings.

## 8 Diagnosis and treatment

According to the 2011 diagnostic criteria for IgG4-RD, the patient presented with gastric and esophageal lesions, elevated serum IgG4, and histopathological findings demonstrating >50 IgG4-positive plasma cells per HPF in gastric body and esophageal tissues with an IgG4/IgG ratio >30%, which supported the diagnosis of IgG4-RD after exclusion of alternative conditions. Although the patient met the 2011 diagnostic criteria for IgG4-RD, classical histopathological features such as storiform fibrosis and obliterative phlebitis were not prominent in gastrointestinal specimens, which is consistent with previous reports indicating that gastrointestinal IgG4-RD may lack typical pathological features. The treatment regimen initially included intravenous methylprednisolone 80 mg daily and cyclophosphamide(CTX) 0.2 g every other day, combined with tamoxifen and esomeprazole. Cyclophosphamide was discontinued due to abnormal liver function during follow-up, and therapy was transitioned to mycophenolate mofetil (MMF)1 g twice daily. The dosage was subsequently tapered to 0.75 g twice daily after 3 months, followed by further reduction to 0.5 g twice daily as maintenance therapy. Follow-up assessments revealed normalization of serum IgG4 levels. Concurrently, methylprednisolone was gradually tapered to a maintenance oral dose of 8 mg daily.

In April 2015, follow-up gastroscopy revealed healed gastric body ulcers and a reduction in esophageal lesion size. However, a repeat abdominal CT scan showed the liver lesion had enlarged to 5.5 cm × 6.2 cm and appeared as a mixed hyper- and hypodense lesion with surrounding patchy hypodense areas. No new liver lesions were observed. The gastric wall at the lower esophagus and gastric fundus along the lesser curvature was markedly thickened. Ultrasound-guided liver biopsy suggested FDCS, though inflammatory pseudotumor could not be entirely excluded, prompting a recommendation for surgical excision to confirm the diagnosis. The patient gradually tapered and discontinued glucocorticoids and immunosuppressants, and underwent resection of the liver lesion in June 2015. Histopathological examination demonstrated a tumor composed of spindle-shaped cells with mild cytological atypia, rare mitotic figures, lymphoplasmacytic infiltration, and extensive areas of degeneration, hemorrhage, and necrosis. Immunohistochemistry supported the diagnosis of FDCS. Immunohistochemical results (**Figure 3**): Ki-67 (+20%), CD3 (scattered positivity), CD20 (abundant positivity), CD138 (scattered positivity), CD21 (+), CD23 (+), S-100 (-), CD35 (+), CD31 (focal positivity), IgG (focal positivity), IgG4 (scattered focal positivity). The oncology team classified the tumor as a low-grade malignancy. Due to uncertain efficacy of radiotherapy or chemotherapy, adjuvant therapy was not recommended postoperatively. The patient was maintained on oral omeprazole 20 mg twice daily.

**Figure 3:**
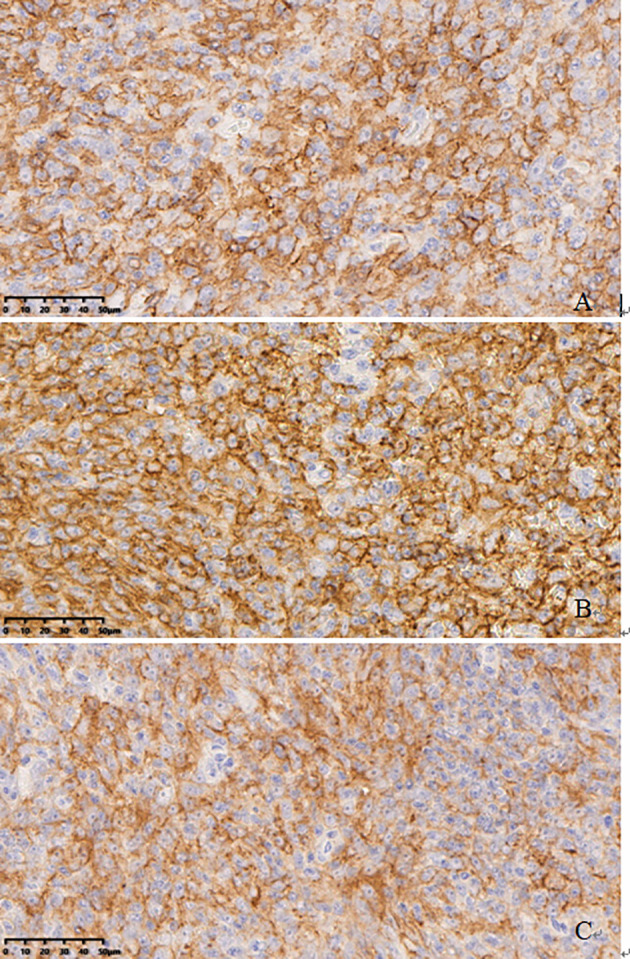
**(A)** CD21 is focally positive in the lesion of liver biopsies. **(B)** CD23 is focally positive in the lesion of liver biopsies. **(C)** CD35 is focally positive in the lesion of liver biopsies.

In January 2016,the patient underwent another follow-up gastroscopy, which revealed a raised lesion with central ulceration in the lower esophagus, ulcerative lesions in the anterior wall and lesser curvature of the gastric body, progression and fusion of the esophageal and gastric lesions, and marked gastric wall rigidity with absent peristalsis.

Pathology (lower esophagus): Squamous epithelial mucosa showed severe acute and chronic inflammation with erosion, ulceration, and inflammatory granuloma formation. There was dense lymphoplasmacytic infiltration and clusters of oval cells with prominent nucleoli and pale chromatin. Combined with immunohistochemistry and clinical history, findings were consistent with FDCS complicated by IgG4-RD. Immunohistochemistry (esophagus): CD21(+), CD138(+), IgG(+, >100 cells/HPF), IgG4(+, >50 cells/HPF).

Pathology (gastric lesser curvature): Proliferative fibrous connective tissue with severe acute and chronic inflammation, erosion, inflammatory granulation tissue, focal necrosis, and dense lymphocytic infiltration. Findings suggested IgG4-RD, with focal features suspicious for coexisting FDCS. Immunohistochemistry (stomach): CD138(+), CD21(focal+), CD23(focal+), CD35(-), CD3(scattered+), CD20(focal+), AE1/AE3(-), Ki-67(+10%), Kappa(+), Lambda(+), IgG(+, >100 cells/HPF), IgG4(+, >50 cells/HPF), EBER(-), CD1a(-), S-100(focal+).

The patient presented with multiple episodes of slow-growing, painless hepatic lesions, ultimately confirmed as hepatic FDCS through histopathological and immunohistochemical analysis. The gastric ulcers had previously healed following treatment with glucocorticoids and immunosuppressive agents; however, after discontinuation of these medications, the gastric ulcers significantly worsened. Based on histopathological and serological findings, a diagnosis of IgG4-related disease involving the esophagus and stomach was established.

The patient was re-treated with oral prednisone 50 mg once daily, cyclophosphamide 100 mg every other day, and omeprazole 20 mg twice daily, with oncology follow-up. In August 2016, abdominal CT revealed enlargement of the lesser curvature gastric lesion, measuring approximately 11.8 cm × 9.7 cm at its largest cross-section, with CT attenuation values ranging from 20 to 36 Hu and persistent marked enhancement. The left renal lesions remained unchanged. Barium meal studies identified multiple occupying lesions in the lower esophagus, gastric fundus, and body, with poor visualization of the duodenal bulb and proximal duodenum. Hepatobiliary surgery recommended discontinuing glucocorticoids prior to surgical intervention, but the patient declined. The patient was discharged without regular follow-up. We contacted the patient’s family by phone. According to the family, the patient died of massive gastrointestinal bleeding, which may have been related to aggravation of gastric ulcer caused by IgG4-RD (**Figure 4**). However, the exact cause and timing of the patient’s death remain unclear, which limits the value of prognostic analysis to some extent.

**Figure 4:**
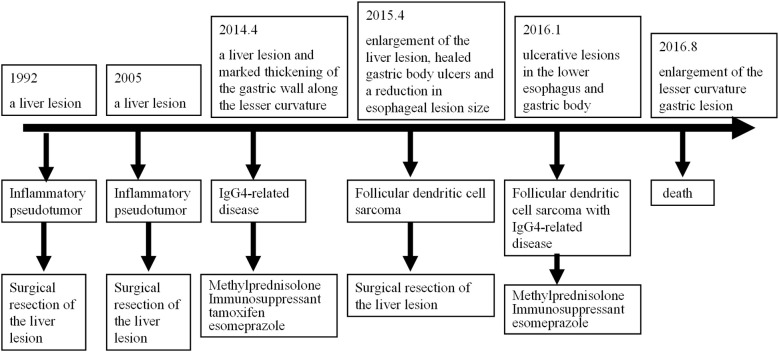
Timeline.

## 9 Discussion and conclusion

FDCS is a rare low-grade malignant tumor that frequently involves lymph nodes but may also affect extranodal sites, including the tonsils, oral cavity, liver, spleen, gastrointestinal tract, lungs, breast, skin, and mediastinum (3). FDCS predominantly occurs in adults with equal gender distribution. Monda et al (4) reported the first case of FDCS in 1986. Cheuk et al (5) reported 11 cases strongly associated with Epstein-Barr virus (EBV) infection, which morphologically resemble inflammatory pseudotumors, leading to the designation inflammatory pseudotumor-like FDCS (IPT-like FDCS). IPT-like FDCS predominantly involves the liver and spleen, though retroperitoneal, gastrointestinal, and tonsillar involvement has also been documented (6). FDCS is a tumor resulting from the malignant transformation of FDCs. Tumor cells retain FDC characteristics and express markers such as CD21 and CD23. When FDCs undergo malignant transformation, FDCS develops. FDCS lacks specific clinical manifestations, typically presenting as slow-growing, painless lesions. Definitive diagnosis relies on histopathological and immunohistochemical analysis, with CD21, CD35, and CD23 recognized as characteristic markers (7). The primary treatment is complete surgical resection, with adjuvant chemotherapy considered in select cases. FDCS exhibits a local recurrence rate of approximately 50% and a distant metastasis rate of 25% (8). Due to its rarity and nonspecific clinical and imaging features, FDCS is frequently misdiagnosed. In our case, the patient presented with recurrent painless hepatic lesions. Histopathological examination revealed that the tumor was composed of spindle cells accompanied by lymphoplasmacytic infiltration and extensive areas of degeneration, hemorrhage, and necrosis. Immunohistochemically, the tumor cells were positive for CD21, CD35, and CD23. A definitive diagnosis of FDCS was established.

IgG4-related disease (IgG4-RD) was first described by Hamano et al (9) in 2001 in the context of autoimmune pancreatitis (AIP) and was formally designated as a distinct clinical entity in 2010 (10). IgG4-RD is a systemic immune-mediated fibrotic disease. The core mechanism involves antigen presentation by B cells to cytotoxic T lymphocytes. Typical pathological features include dense lymphoplasmacytic infiltration, predominantly composed of T cells, B cells, eosinophils, and plasma cells, with prominent extranodal germinal centers often observed (11).IgG4-RD exhibits diverse clinical manifestations and can affect nearly all organ systems, with typical involvement of the pancreas, salivary glands, bile ducts, orbits, kidneys, lungs, aorta, retroperitoneum, meninges, or thyroid. IgG4-RD can also involve the entire gastrointestinal tract, including the esophagus, stomach, duodenum, small intestine, and colon; however, it rarely involves the stomach and esophagus in isolation (12). Literature reports indicate that esophageal involvement in IgG4-related disease (IgG4-RD) may manifest with nausea, vomiting, dysphagia, and retrosternal pain (13, 14). Endoscopic findings include esophagitis, esophageal ulcers, nodules, lesion-like protrusions, or luminal elevations (15, 16). Uehara et al. reviewed 39 cases of IgG4-RD with gastric involvement, finding that only 40% presented with typical gastric manifestations. The majority of gastric lesions manifested as inflammatory tumors, ulcers, nodular lesions, areas of chronic gastritis, and malignancy (17).

The current diagnosis of IgG4-RD primarily relies on the 2011 Japanese comprehensive diagnostic criteria for IgG4-RD, its 2020 revised version, and the Consensus Statement on the Pathology of IgG4-RD (18–20). However, elevated serum IgG4 is not a disease-specific biomarker for IgG4-RD, as it may also occur in diverse conditions including diffuse connective tissue diseases, malignancies, chronic infections, and allergic disorders. Furthermore, not all IgG4-RD patients exhibit elevated serum IgG4. Consequently, the 2019 *International Classification Criteria for IgG4-RD* jointly established by ACR and EULAR (2)emphasize the characteristic clinical or radiological features of typically involved organs and incorporate exclusion criteria, thereby enhancing diagnostic specificity. These criteria are particularly suited for clinical research in IgG4-RD. Their notable advantage lies in enabling the classification of patients as IgG4-RD even in the absence of pathological confirmation or normal serum IgG4 levels. In this case, focal IgG4-positive plasma cells (>50/HPF) and an IgG4/IgG ratio>30% were observed, which do not meet the 2011 Japanese comprehensive diagnostic criteria for IgG4-related diseases (IgG4-RD). However, the number of IgG4-positive plasma cells per high-power field varies across different tissues, and the incidence of storiform fibrosis and obliterative phlebitis also varies. Given the rarity of IgG4-RD involving the esophagus, stomach, and intestines, the European Federation of Gastroenterology (UEG) and the Swedish Society of Gastroenterology (SGF) jointly developed guidelines for IgG4-related digestive diseases in June 2020 (21). The guidelines state that IgG4-RD involving the esophagus, stomach, and intestines is rare or nonexistent. For gastrointestinal lesions with unclear/non-diagnostic histology and negative findings for malignancy, empirical glucocorticoid treatment for one month may be an appropriate option. In this case, other diseases have been ruled out. Based on serological tests, pathological findings, and response to glucocorticoid therapy, we still consider that IgG4-RD involves the esophagus and stomach. Organ involvement of the esophagus, stomach, and intestinal tract in IgG4-RD remains subject to substantial diagnostic uncertainty. IgG4-RD often involves multiple organ systems, necessitating multidisciplinary collaboration. However, due to under-recognition, misdiagnosis and delayed diagnosis remain prevalent. Early intervention with glucocorticoids significantly improves prognosis, highlighting the need for heightened clinical awareness and prompt diagnosis.

Through a comprehensive literature review, we compiled 44 cases of FDCS with increased IgG4-positive plasma cells (22–29)(**Table 1**). The cohort included 16 male and 28 female patients, with a male-to-female ratio of 4:7. The patients ranged from 21 to 84 years old, with a mean age of 51.1 years. Tumor sites included the liver (n=11, 25%), spleen (n=20, 45.5%), colon polyps (n=4, 9.1%), retroperitoneal lesion (n=3, 6.8%), and one case each in the lung, right adrenal gland, left supraclavicular lesion, left axillary lesion, left neck lesion, and right inguinal lymph node(n=1, 2.3%). Immunohistochemical analysis revealed IgG4-positive plasma cell counts ranging from 1 to 240 cells/HPF, with IgG4/IgG ratios varying from not available to 83%. Epstein-Barr virus (EBV) status was positive in 35 cases(79.5%), negative in 8 cases(18.2%), and not reported (NA) in 1case. Serum IgG4 levels were elevated in only one patient. Most reported cases were from retrospective studies in which serum IgG4 levels were not routinely measured, and some cases had inaccurate IgG4-positive cell counts. These limitations affected the comparative analysis. FDCS accompanied by an increase in IgG4-positive plasma cells is predominantly located in the spleen, liver, and colon, and approximately four-fifths of cases are associated with Epstein-Barr virus (EBV) infection. EBV positivity in tumor cells is one of the key diagnostic features of FDCS, but it is not absolute. A literature review showed that 79.5% of tested cases were EBV-positive. A case of EBV-negative FDCS involving the liver was reported (29). In patients with EBV-positive IPT-like FDCS, the expression levels of IgG4, EBER, and SMA are significantly higher than those in patients with conventional FDCS, whereas the expression levels of SSTR2, EGFR, and STAT3 are significantly lower (25). Four patients with EBV-positive IPT-like FDCS had genetic mutations, and further research revealed that cAMP plays a key role in tumor development. This also supports, from another perspective, that EBV negativity in our patient is plausible. EBV-negative IPT-like FDCS may represent a unique subgroup with distinct clinicopathological characteristics. This finding highlights the need for larger case series to better characterize the disease spectrum of FDCS and to explore whether EBV status is associated with clinical outcomes, treatment response, or concomitant diseases such as IgG4-RD.

**Table 1:** Clinical characteristics of FDCS with increased IgG4-positive plasma cells.

Case	Reference	Gender	Age	Site of involvement	IgG4 (+cells/HPF)	IgG4/IgG ratio(%)	EBV	serum IgG4	Treatment	Outcome
1	Li Y etal. (2023) (22)	M	45	Liver	60	24	+	NA	Lobectomy of liver and chemotherapy	19 months, Recurrence
2	Li Y etal. (2023) (22)	M	55	Liver	6	3	+	NA	Lobectomy of liver	15 months, Recurrence
3	Li Y etal. (2023) (22)	M	48	Liver	110	44	+	NA	Lobectomy of liver	76 months,AWD
4	Li Y etal. (2023) (22)	M	58	Liver	30	30	+	NA	Lobectomy of liver	8 months,AWD
5	Li Y etal. (2023) (22)	F	48	Liver	2	1	+	NA	Lobectomy of liver	6 months,AWD
6	Li Y etal. (2023) (22)	F	64	Spleen	30	30	+	NA	Splenectomy	36 months,AWD
7	Li Y etal. (2023) (22)	F	61	Spleen	100	83	+	NA	Splenectomy	78 months,AWD
8	Li Y etal. (2023) (22)	F	67	Spleen	35	18	+	NA	Splenectomy	7 months,AWD
9	Li Y etal. (2023) (22)	M	34	Liver	1	20	+	NA	Lobectomy of liver	55 months,AWD
10	Li Y etal. (2023) (22)	F	49	Spleen	20	50	+	NA	Splenectomy	12 months, AWD
11	Li Y etal. (2023) (22)	F	29	Spleen	40	80	+	NA	Splenectomy	NA
12	Li Y etal. (2023) (22)	M	38	Liver	20	29	+	NA	Lobectomy of liver	1 month,AWD
13	Li Y etal. (2023) (22)	F	51	Lung	8	10	+	NA	Lobectomy of lung	2 months,AWD
14	Lu Y etal. (2020) (23)	M	16	Right adrenal	2	7	–	NA	Surgical resection	42 months, No Recurrence
15	Lu Y etal. (2020) (23)	F	45	Left supraclavicular lesion	2	8	–	NA	Surgical resection, CHOP chemotherapy 6 months	72 months, No Recurrence
16	Lu Y etal. (2020) (23)	F	37	Left oxter lesion	4	8	–	NA	Surgical resection	Loss
17	Lu Y etal. (2020) (23)	F	41	Retroperitoneal lesion	1	5	–	NA	Surgical resection	Loss
18	Lu Y etal. (2020) (23)	F	33	Retroperitoneal lesion	1	2	+	NA	Surgical resection	8 months, No Recurrence
19	Lu Y etal. (2020) (23)	M	35	Retroperitoneal lesion	4	5	–	NA	Surgical resection, CHOP chemotherapy 2 courses	38 months, bone metastases
20	Lu Y etal. (2020) (23)	M	53	Left neck lesion	2	1	–	NA	Surgical resection	Loss
21	Lu Y etal. (2020) (23)	F	45	Right inguinal lymph node	1	4	–	NA	Surgical resection, CHOP chemotherapy 7 courses	7 months, No Recurrence
22	Lu Y etal. (2020) (23)	M	39	Liver	1	3	NA	NA	Surgical resection	41 months, No Recurrence
23	Choe JY etal. (2013) (24)	F	64	Spleen	27	25	++	NA	Splenectomy	78 months,alive
24	Choe JY etal. (2013) (24)	F	72	Spleen	128	75	++	NA	Splenectomy	18 months,alive
25	Choe JY etal. (2013) (24)	F	53	Spleen	78	50	++	NA	Splenectomy	13 months,alive
26	Choe JY etal. (2013) (24)	M	76	Spleen	58	40	++	NA	Splenectomy	8 months,alive
27	Choe JY etal. (2013) (24)	M	72	Spleen	45	30	++	NA	Splenectomy	18 months,alive
28	Choe JY etal. (2013) (24)	M	75	Spleen	105	60	++	NA	Splenectomy	30 months,alive
29-40	Siqi Chen etal. (2024) (25)	M(2) F(10)	21-84	Spleen(8), liver(2), colon(2)	3-51	3-62	+	NA	NA	2cases,Recurrence
41	Nie C etal. (2024) (26)	F	59	Spleen	a minimal proportion	NA	+	NA	NA	alive
42	Hu J etal. (2023) (27)	F	52	colon polyp	>100	40~50	+	NA	Endoscopic polypectomy	15 months, No Recurrence
43	Goh L etal. (2020) (28)	F	46	colon polyp	240	70	+	raised(2.08 g/L)	colectomy	5 months, No Recurrence
44	Liu X etal. (2021) (29)	M	61	liver	less than10	NA	–	NA	Hepatic partial caudatectomy	13 months, No Recurrence
45	Our case	M	60	Liver, esophagus, stomach	>50 in esophagus and stomach	>30 in esophagu-s and stomach	–	raised(1590 mg/L)	Surgical resection, Glucocorticoids and immunosuppressants	death

AWD indicates alive without disease; F, female; M, male; NA, not available.

The overlapping histopathological features between IPT-like FDCS and IgG4-RD, particularly IgG4-positive plasma cell infiltration, represent a major diagnostic pitfall. The coexistence of FDCS and IgG4-RD in this case raises the possibility of a shared immunological mechanism. FDCs are mesenchymal-derived dendritic cells located in B-cell follicles. They play a key role in initiating and maintaining B-cell adaptive immune responses. FDCS predominantly occurs in extranodal sites (79.4%) and lymph nodes (15.1%) (1). Due to its variable growth patterns and cytological features, FDCS is easily confused with various tumors and even inflammatory processes. Therefore, a broad panel of follicular dendritic cell markers (including CD21, CD35, and CD23) is required for diagnosis. In addition to distinct histopathological and clinical characteristics, EBV positivity in tumor cells is also a key diagnostic feature of FDCS. The relationship between these two entities centers on FDCs. FDCs are not tumor cells but are important components of the immune system. Their primary function is to capture antigens and present them to B cells, and they play a key role in the formation of tertiary lymphoid organs (TLOs). Studies have shown that abundant TLOs are present in the affected tissues of patients with IgG4-RD. Within these structures, FDCs form a dense “mesh scaffold” that recruits and promotes interactions between follicular helper T cells (Tfh) and CXCR5-expressing B cells by secreting the chemokine CXCL13 (30). This interaction promotes the differentiation of B cells into plasma cells that produce large amounts of IgG4 and drives the disease toward fibrosis. Therefore, FDC involvement is a key factor in the persistence and amplification of the inflammatory response in IgG4-RD.

The differential diagnosis of this case requires focused attention on the following aspects: Differentiation of hepatic FDCS: The core challenge lies in distinguishing it from other intrahepatic spindle cell tumors, carcinomas, and lymphomas. Immunohistochemical detection of CD21/CD23/CD35 is the key.

Differentiation of IgG4-RD involving the esophagus and stomach: It is essential to exclude peptic ulcer disease, malignant tumors, and MALT lymphoma. The number of IgG4-positive plasma cells and the IgG4/IgG ratio on pathology are the core indicators.

Differentiation when both conditions coexist: Caution is required to avoid misinterpreting reactive IgG4-positive plasma cell infiltration in FDCS as IgG4-RD, and vice versa. Comprehensive immunohistochemical profiling (including FDC markers, IgG4/IgG ratio, and EBER) combined with clinical treatment response is critical for accurate diagnosis.

In conclusion, we reported the first case of IPT-like FDCS associated with IgG4-related disease involving the liver and gastrointestinal tract. The significant overlap in histopathological features between these entities poses diagnostic challenges. Accurate diagnosis requires integration of clinical, imaging, and immunohistochemical findings. Increased awareness of this rare association may reduce misdiagnosis. This case underscores the importance of maintaining a high index of suspicion for malignancy in patients with presumed IgG4-RD who exhibit atypical clinical progression or poor response to therapy. As a single case report, our results are primarily intended to generate hypotheses rather than draw definitive conclusions. Further studies are required to clarify the pathogenic relationship between FDCS and IgG4-RD and to determine the clinical significance of IgG4-positive plasma cell infiltration in FDCS.

## Data Availability

The original contributions presented in the study are included in the article/supplementary material. Further inquiries can be directed to the corresponding author.

## References

[1] Facchetti F Simbeni M Lorenzi L . Follicular dendritic cell sarcoma. Pathologica. (2021) 113:316–29. doi: 10.32074/1591-951X-331 34837090 PMC8720404

[2] Wallace ZS Naden RP Chari S Choi H Della-Torre E Dicaire JF . The 2019 American college of rheumatology/European league against rheumatism classification criteria for igG4-related disease. Arthritis Rheumatol. (2020) 72:7–19. doi: 10.1002/art.41120 31793250

[3] Ohtake H Yamakawa M . Interdigitating dendritic cell sarcoma and follicular dendritic cell sarcoma: histopathological findings for differential diagnosis. J Clin Exp Hematop. (2013) 53:179–84. doi: 10.3960/jslrt.53.179 24369219

[4] Monda L Warnke R Rosai J . A primary lymph node Malignancy with features suggestive of dendritic reticulum cell differentiation. A report of 4 cases. Am J Pathol. (1986) 122:562–72. PMC1888214 2420185

[5] Cheuk W Chan JK Shek TW Chang JH Tsou MH Yuen NW . Inflammatory pseudotumor-like follicular dendritic cell tumor: a distinctive low-grade Malignant intra-abdominal neoplasm with consistent Epstein-Barr virus association. Am J Surg Pathol. (2001) 25:721–31. doi: 10.1097/00000478-200106000-00003 11395549

[6] Jiang XN Zhang Y Xue T Chen JY Chan ACL Cheuk W . New clinicopathologic scenarios of EBV+ inflammatory follicular dendritic cell sarcoma: report of 9 extrahepatosplenic cases. Am J Surg Pathol. (2021) 45:765–72. doi: 10.1097/PAS.0000000000001632 33264138

[7] Patra S Trivedi P Kaur K . Histiocytic and follicular dendritic cell sarcoma: diagnostically challenging rare entities. Indian J Pathol Microbiol. (2021) 64:316–22. doi: 10.4103/IJPM.IJPM_461_20 33851626

[8] Wu A Pullarkat S . Follicular dendritic cell sarcoma. Arch Pathol Lab Med. (2016) 140:186–90. doi: 10.5858/arpa.2014-0374-RS 26910224

[9] Hamano H Kawa S Horiuchi A Unno H Furuya N Akamatsu T . High serum IgG4 concentrations in patients with sclerosing pancreatitis. N Engl J Med. (2001) 344:732–8. doi: 10.1056/NEJM200103083441005 11236777

[10] Takahashi H Yamamoto M Suzuki C Naishiro Y Shinomura Y Imai K . The birthday of a new syndrome: IgG4-related diseases constitute a clinical entity. Autoimmun Rev. (2010) 9:591–4. doi: 10.1016/j.autrev.2010.05.003 20457280

[11] Stone JH . IgG4-related disease: lessons from the first 20 years. Rheumatol (Oxford). (2025) 64:i24–7. doi: 10.1093/rheumatology/keaf008 40071397

[12] Wallace ZS Zhang Y Perugino CA Naden R Choi HK Stone JH . Clinical phenotypes of IgG4-related disease: an analysis of two international cross-sectional cohorts. Ann Rheum Dis. (2019) 78:406–12. doi: 10.1136/annrheumdis-2018-214603 30612117 PMC6996288

[13] Jang SW Jeon MH Shin HD . IgG4-related disease with esophageal involvement. Case Rep Gastroenterol. (2019) 13:369–75. doi: 10.1159/000502794 31607838 PMC6787435

[14] Padniewski JJ Thottam GE Nasr R . IgG4 sclerosing disease of the esophagus: a case-based review. Rheumatol Int. (2020) 40:1733–7. doi: 10.1007/s00296-020-04594-5 32358641

[15] Mine S Ozawa E Ohnita K Abe K Nakao K . A case of IgG4-related disease presenting with multiple gastric submucosal tumors. Gastrointest Endosc. (2018) 87:608–10. doi: 10.1016/j.gie.2017.07.010 28720475

[16] Vashi B Khosroshahi A . IgG4-related disease with emphasis on its gastrointestinal manifestation. Gastroenterol Clin North Am. (2019) 48:291–305. doi: 10.1016/j.gtc.2019.02.008 31046976

[17] Uehara T Hamano H Kawa S Sano K Oki K Kobayashi Y . Chronic gastritis in the setting of autoimmune pancreatitis. Am J Surg Pathol. (2010) 34:1241–9. doi: 10.1097/PAS.0b013e3181ec07ee 20697253

[18] Umehara H Okazaki K Masaki Y Kawano M Yamamoto M Saeki T . Comprehensive diagnostic criteria for IgG4-related disease (IgG4-RD), 2011. Mod Rheumatol. (2012) 22:21–30. doi: 10.1007/s10165-011-0571-z 22218969

[19] Umehara H Okazaki K Kawa S Takahashi H Goto H Matsui S . The 2020 revised comprehensive diagnostic (RCD) criteria for IgG4-RD. Mod Rheumatol. (2021) 31:529–33. doi: 10.1080/14397595.2020.1859710 33274670

[20] Deshpande V Zen Y Chan JK Yi EE Sato Y Yoshino T . Consensus statement on the pathology of IgG4-related disease. Mod Pathol. (2012) 25:1181–92. doi: 10.1038/modpathol.2012.72 22596100

[21] Löhr JM Beuers U Vujasinovic M Alvaro D Frøkjær JB Buttgereit F . European guideline on IgG4-related digestive disease - UEG and SGF evidence-based recommendations. United Eur Gastroenterol J. (2020) 8:637–66. doi: 10.1177/2050640620934911 32552502 PMC7437085

[22] Li Y Yang X Tao L Zeng W Zuo M Li S . Challenges in the diagnosis of Epstein-Barr virus-positive inflammatory follicular dendritic cell sarcoma: extremely wide morphologic spectrum and immunophenotype. Am J Surg Pathol. (2023) 47:476–89. doi: 10.1097/PAS.0000000000002011 36574358

[23] Lu Y Liu QL Lu T Pang JY Huo Z . Clinicopathological features of follicular dendritic cell sarcoma. Zhongguo Yi Xue Ke Xue Yuan Xue Bao. (2020) 42:504–12. doi: 10.3881/j.issn.1000-503X.11785 32895103

[24] Choe JY Go H Jeon YK Yun JY Kim YA Kim HJ . Inflammatory pseudotumor-like follicular dendritic cell sarcoma of the spleen: a report of six cases with increased IgG4-positive plasma cells. Pathol Int. (2013) 63:245–51. doi: 10.1111/pin.12057 23714251

[25] Chen S You Z Chen X Wang C . Clinicopathological and molecular genetic insights into EBV-positive inflammatory follicular dendritic cell sarcoma. Hum Pathol. (2024) 153:105668. doi: 10.1016/j.humpath.2024.105668 39370049

[26] Nie C Xie X Li H Li Y Chen Z Li Y . Epstein-Barr virus-positive inflammatory follicular dendritic cell sarcoma with significant granuloma: case report and literature review. Diagn Pathol. (2024) 19:34. doi: 10.1186/s13000-024-01457-6 38365739 PMC10870656

[27] Hu J Huang D Xu C Chen Y Ma H Shen Z . Epstein-Barr virus-positive inflammatory follicular dendritic cell sarcoma presenting as a colonic polyp: report of a case with a literature review. Med (Kaunas). (2023) 59:1341. doi: 10.3390/medicina59071341 37512154 PMC10385426

[28] Goh L Teo NZ Wang LM . Beware the inflammatory cell-rich colonic polyp: a rare case of EBV-positive inflammatory pseudotumour-like follicular dendritic cell sarcoma with increased IgG4-positive plasma cells. Pathology. (2020) 52:713–7. doi: 10.1016/j.pathol.2020.05.011 32814623

[29] Liu X Cao L Chin W Yu J Liu Y Zheng S . Epstein-Barr virus-negative inflammatory pseudotumor-like variant of follicular dendritic cell sarcoma of the liver: a case report and literature review. Clin Res Hepatol Gastroenterol. (2021) 45:101457. doi: 10.1016/j.clinre.2020.05.007 32540141

[30] Xu J Zhai J Zhao J . Pathogenic roles of follicular helper T cells in IgG4-related disease and implications for potential therapy. Front Immunol. (2024) 15:1413860. doi: 10.3389/fimmu.2024.1413860 38911857 PMC11190345

