# Correction: Follicular dendritic cell sarcoma associated with IgG4-related disease: a case report and literature review

**DOI:** 10.3389/fimmu.2026.1905419

**Published:** 2026-06-26

**Authors:** Yajuan Yao, Yunjing Bai, Guang Liu, Yu Du, Xiaoyu Long, Xiaohua Xu, Fan Wu, Xiaorong Jin

**Affiliations:** 1 Department of Rheumatism and Immunology, Seventh Medical Center of Chinese PLA General Hospital, Beijing, China; 2 Department of Pathology, Seventh Medical Center of Chinese PLA General Hospital, Beijing, China

**Keywords:** esophagus, follicular dendritic cell sarcoma, gastric, IgG4-related disease, liver

There was a mistake in **Table 1** as published. “A post-publication self-check confirmed a series of sequential reference number errors in **Table 1** (Clinical characteristics of FDCS with increased IgG4-positive plasma cells)”. These errors occurred due to our oversight in the final proofreading stage, for which we take full responsibility. The full list of corrections for the Reference column of **Table 1** is shown below:

**TABLE 1:** Clinical characteristics of FDCS with increased IgG4-positive plasma cells.

Case	Reference	Gender	Age	Site of involvement	IgG4 (+cells/HPF)	IgG4/IgG ratio(%)	EBV	serum IgG4	Treatment	Outcome
1	Li Y etal. (2023)^[22]^	M	45	Liver	60	24	+	NA	Lobectomy of liver and chemotherapy	19 months, Recurrence
2	Li Y etal. (2023)^[22]^	M	55	Liver	6	3	+	NA	Lobectomy of liver	15 months, Recurrence
3	Li Y etal. (2023)^[22]^	M	48	Liver	110	44	+	NA	Lobectomy of liver	76 months, AWD
4	Li Y etal. (2023)^[22]^	M	58	Liver	30	30	+	NA	Lobectomy of liver	8 months, AWD
5	Li Y etal. (2023)^[22]^	F	48	Liver	2	1	+	NA	Lobectomy of liver	6 months, AWD
6	Li Y etal. (2023)^[22]^	F	64	Spleen	30	30	+	NA	Splenectomy	36 months, AWD
7	Li Y etal. (2023)^[22]^	F	61	Spleen	100	83	+	NA	Splenectomy	78 months, AWD
8	Li Y etal. (2023)^[22]^	F	67	Spleen	35	18	+	NA	Splenectomy	7 months, AWD
9	Li Y etal. (2023)^[22]^	M	34	Liver	1	20	+	NA	Lobectomy of liver	55 months, AWD
10	Li Y etal. (2023)^[22]^	F	49	Spleen	20	50	+	NA	Splenectomy	12 months, AWD
11	Li Y etal. (2023)^[22]^	F	29	Spleen	40	80	+	NA	Splenectomy	NA
12	Li Y etal. (2023)^[22]^	M	38	Liver	20	29	+	NA	Lobectomy of liver	1 month, AWD
13	Li Y etal. (2023)^[22]^	F	51	Lung	8	10	+	NA	Lobectomy of lung	2 months, AWD
14	Lu Y etal. (2020)^[23]^	M	16	Right adrenal	2	7	–	NA	Surgical resection	42 months, No Recurrence
15	Lu Y etal. (2020)^[23]^	F	45	Left supraclavicular lesion	2	8	–	NA	Surgical resection, CHOP chemotherapy 6 months	72 months, No Recurrence
16	Lu Y etal. (2020)^[23]^	F	37	Left oxter lesion	4	8	–	NA	Surgical resection	Loss
17	Lu Y etal. (2020)^[23]^	F	41	Retroperitoneal lesion	1	5	–	NA	Surgical resection	Loss
18	Lu Y etal. (2020)^[23]^	F	33	Retroperitoneal lesion	1	2	+	NA	Surgical resection	8 months, No Recurrence
19	Lu Y etal. (2020)^[23]^	M	35	Retroperitoneal lesion	4	5	–	NA	Surgical resection, CHOP chemotherapy 2 courses	38 months, bone metastases
20	Lu Y etal. (2020)^[23]^	M	53	Left neck lesion	2	1	–	NA	Surgical resection	Loss
21	Lu Y etal. (2020)^[23]^	F	45	Right inguinal lymph node	1	4	–	NA	Surgical resection, CHOP chemotherapy 7 courses	7 months, No Recurrence
22	Lu Y etal. (2020)^[23]^	M	39	Liver	1	3	NA	NA	Surgical resection	41 months, No Recurrence
23	Choe JY etal. (2013)^[24]^	F	64	Spleen	27	25	++	NA	Splenectomy	78 months, alive
24	Choe JY etal. (2013)^[24]^	F	72	Spleen	128	75	++	NA	Splenectomy	18 months, alive
25	Choe JY etal. (2013)^[24]^	F	53	Spleen	78	50	++	NA	Splenectomy	13 months, alive
26	Choe JY etal. (2013)^[24]^	M	76	Spleen	58	40	++	NA	Splenectomy	8 months, alive
27	Choe JY etal. (2013)^[24]^	M	72	Spleen	45	30	++	NA	Splenectomy	18 months, alive
28	Choe JY etal. (2013)^[24]^	M	75	Spleen	105	60	++	NA	Splenectomy	30 months, alive
29-40	Siqi Chen etal. (2024)^[25]^	M(2) F(10)	21-84	Spleen(8), liver(2), colon(2)	3-51	3-62	+	NA	NA	2cases, Recurrence
41	Nie C etal. (2024)^[26]^	F	59	Spleen	a minimal proportion	NA	+	NA	NA	alive
42	Hu J etal. (2023)^[27]^	F	52	colon polyp	>100	40~50	+	NA	Endoscopic polypectomy	15 months, No Recurrence
43	Goh L etal. (2020)^[28]^	F	46	colon polyp	240	70	+	raised(2.08 g/L)	colectomy	5 months, No Recurrence
44	Liu X etal. (2021)^[29]^	M	61	liver	less than10	NA	–	NA	Hepatic partial caudatectomy	13 months, No Recurrence
45	Our case	M	60	Liver, esophagus, stomach	>50 in esophagus and stomach	>30 in esophagus and stomach	–	raised(1590 mg/L)	Surgical resection, Glucocorticoids and immunosuppressants	death

AWD indicates alive without disease; F, female; M, male; NA, not available.

1. “Li Y etal. (2023) (21)” should be revised to “Li Y etal. (2023) (22)”
2. “Lu Y etal. (2020) (22)” should be revised to “Lu Y etal. (2020) (23)”
3. “Choe JY etal. (2013) (23)” should be revised to “Choe JY etal. (2013) (24)”
4. “Siqi Chen etal. (2024) (24)” should be revised to “Siqi Chen etal. (2024) (25)”
5. “Nie C etal. (2024) (25)” should be revised to “Nie C etal. (2024) (26)”
6. “Hu J etal. (2023) (26)” should be revised to “Hu J etal. (2023) (27)”
7. “Goh L etal. (2020) (27)” should be revised to “Goh L etal. (2020) (28)”
8. “Liu X etal. (2021) (28)” should be revised to “Liu X etal. (2021) (29)”.

The corrected **Table 1** appears below.

The original version of this article has been updated.

